# Identification of the hybrid gene *LILRB5-3* by long-read sequencing and implication of its novel signaling function

**DOI:** 10.3389/fimmu.2024.1398935

**Published:** 2024-05-14

**Authors:** Kouyuki Hirayasu, Seik-Soon Khor, Yosuke Kawai, Mihoko Shimada, Yosuke Omae, Gen Hasegawa, Yuko Hashikawa, Hiromu Tanimoto, Jun Ohashi, Kazuyoshi Hosomichi, Atsushi Tajima, Hiroyuki Nakamura, Minoru Nakamura, Katsushi Tokunaga, Rikinari Hanayama, Masao Nagasaki

**Affiliations:** ^1^ Advanced Preventive Medical Sciences Research Center, Kanazawa University, Kanazawa, Japan; ^2^ Department of Evolutionary Immunology, Graduate School of Advanced Preventive Medical Sciences, Kanazawa University, Kanazawa, Japan; ^3^ Department of Immunology, Graduate School of Medical Sciences, Kanazawa University, Kanazawa, Japan; ^4^ Department of Immunology, School of Medical and Pharmaceutical Sciences, Kanazawa University, Kanazawa, Japan; ^5^ Genome Medical Science Project, Research Institute, National Center for Global Health and Medicine, Tokyo, Japan; ^6^ Singapore Centre for Environmental Life Sciences Engineering, Nanyang Technological University, Singapore, Singapore; ^7^ WPI Nano Life Science Institute (NanoLSI), Kanazawa University, Kanazawa, Japan; ^8^ Department of Biological Sciences, Graduate School of Science, The University of Tokyo, Tokyo, Japan; ^9^ Laboratory of Computational Genomics, School of Life Science, Tokyo University of Pharmacy and Life Sciences, Tokyo, Japan; ^10^ Department of Bioinformatics and Genomics, Graduate School of Advanced Preventive Medical Sciences, Kanazawa University, Kanazawa, Japan; ^11^ Department of Hygiene and Public Health, Faculty of Medicine, Institute of Medical, Pharmaceutical and Health Sciences, Kanazawa University, Kanazawa, Japan; ^12^ Clinical Research Center, National Hospital Organization (NHO) Nagasaki Medical Center, Omura, Japan; ^13^ Department of Hepatology, Nagasaki University Graduate School of Biomedical Sciences, Omura, Japan; ^14^ Headquarters of Primary Biliary Cholangitis (PBC) Research in NHO Study Group for Liver Disease in Japan (NHOSLJ), Clinical Research Center, NHO Nagasaki Medical Center, Omura, Japan; ^15^ Division of Biomedical Information Analysis, Medical Research Center for High Depth Omics, Medical Institute of Bioregulation, Kyushu University, Fukuoka, Japan; ^16^ Center for Genomic Medicine, Graduate School of Medicine, Kyoto University, Kyoto, Japan

**Keywords:** LILR, LILRB3, LILRA6, LILRB5, deletion, long-read sequencing, copy number variation, inhibitory receptor

## Abstract

Leukocyte immunoglobulin (Ig)-like receptors (LILRs) on human chromosome 19q13.4 encode 11 immunoglobulin superfamily receptors, exhibiting genetic diversity within and between human populations. Among the *LILR* genes, the genomic region surrounding *LILRB3* and *LILRA6* has yet to be fully characterized due to their significant sequence homology, which makes it difficult to differentiate between them. To examine the *LILRB3* and *LILRA6* genomic region, a tool named JoGo-LILR CN Caller, which can call copy number from short-read whole genome sequencing (srWGS) data, was applied to an extensive international srWGS dataset comprising 2,504 samples. During this process, a previously unreported loss of both *LILRB3* and *LILRA6* was detected in three samples. Using long-read sequencing of these samples, we have discovered a novel large deletion (33,692 bp) in the *LILRB3* and *LILRA6* genomic regions in the Japanese population. This deletion spanned three genes, *LILRB3*, *LILRA6*, and *LILRB5*, resulting in *LILRB3* exons 12-13 being located immediately downstream of *LILRB5* exons 1-12 with the loss of *LILRA6*, suggesting the potential expression of a hybrid gene between *LILRB5* and *LILRB3* (*LILRB5-3*). Transcription and subsequent translation of the *LILRB5-3* hybrid gene were also verified. The hybrid junction was located within the intracellular domain, resulting in an LILRB5 extracellular domain fused to a partial LILRB3 intracellular domain with three immunoreceptor tyrosine-based inhibitory motifs (ITIMs), suggesting that LILRB5-3 acquired a novel signaling function. Further application of the JoGo-LILR tool to srWGS samples suggested the presence of the *LILRB5-3* hybrid gene in the CEU population. Our findings provide insight into the genetic and functional diversity of the LILR family.

## 1 Introduction

Leukocyte immunoglobulin-like receptors (LILRs), which belong to the immunoglobulin superfamily, are found in primates, but not in rodents, which have an analogous receptor system such as paired Ig-like receptors (PIR) (1). The human *LILR* multigene family is encoded by human chromosome 19q13.4, and consists of five inhibitory LILRBs (LILRB1, LILRB2, LILRB3, LILRB4, and LILRB5), five activating LILRAs (LILRA1, LILRA2, LILRA4, LILRA5, and LILRA6), one secretory protein LILRA3, and two pseudogenes (*LILRP1* and *LILRP2*). Inhibitory LILRs possess a long cytoplasmic domain containing immunoreceptor tyrosine-based inhibitory motifs (ITIMs) for the transmission of inhibitory signals. In contrast, activating LILRs possess a positively charged arginine residue in their transmembrane domains that associates with the FcRγ-chain, which contains immunoreceptor tyrosine-based activating motifs (ITAM) for the transmission of activation signals. LILRs recognize both endogenous and exogenous ligands. For instance, LILRB1 and LILRB2 can recognize self-molecules, such as human leukocyte antigen (HLA) class I molecules (2–4), potentially preventing self-responses. In particular, LILRB1 recognizes the conserved α3 domain of HLA class I in a β2-microglobulin (β2m) -dependent manner, while LILRB2 additionally exhibits recognition of the β2m-free heavy chain of HLA class I molecules (5). In either way, the recognition site is different from that of T cell receptors (TCRs), which specifically recognize the α1 and α2 domains of HLA class I. However, the inhibitory functions of LILRBs can be exploited by pathogens for immune evasion through the recognition of exogenous ligands from pathogens (6, 7). Currently, the role of LILRA activation is not fully understood. One potential function may be the recognition of non-self-ligands for immune activation, as demonstrated by LILRA2, which recognizes microbially cleaved immunoglobulins to detect invading pathogens (8). The expression patterns of the LILR family vary by gene and are found not only in immune cells but also in non-immune cells, such as neurons (9), suggesting that the LILR family may also play important roles in non-immune functions.

Numerous genetic variants have been discovered in *LILR* genes thus far (10). *LILRB1* and *LILRB2* have moderately non-synonymous single nucleotide polymorphisms (SNPs), whereas *LILRB4*, *LILRB5*, *LILRA1*, *LILRA2*, *LILRA4*, and *LILRA5* appear to be relatively conserved with some functional SNPs. *LILRA3* has null alleles detected exclusively in Northeast Asians (11, 12). Among the *LILR* genes, *LILRB3* and *LILRA6* exhibit the highest allelic diversity. In pairwise comparisons, *LILRB3* and *LILRA6* alleles exhibit significantly elevated ratios of non-synonymous to synonymous substitutions, indicating natural selection (13–16). Genome-wide association studies have revealed several associations with the *LILR* genomic region, such as serum levels of creatine kinase and lactate dehydrogenase (*LILRB5*) (17, 18), prostate cancer (*LILRA3*) (19), and Takayasu arteritis (*LILRB3*/*LILRA3*) (20, 21).

The *LILR* genomic region also exhibits extensive copy number variation (CNV). *LILRA3* has 0-2 copies per individual (22), and is one of the most differentiated genes in the human genome (11, 12, 23). *LILRA3* CNV can be explained by a single 6.7 kb deletion. In contrast, *LILRA6* displays a broad range of CNVs, ranging from 0 - 6 copies per individual, indicating the presence of both gene duplications and deletions (13–15, 23). However, the genomic structure of the *LILRA6* gene has not yet been fully elucidated. *LILRA6* shares a high degree of homology with the adjacent gene *LILRB3* within their shared 5 kbp region. Consequently, short-read sequencing technology cannot distinguish between *LILRA6* and *LILRB3* due to the long-range homologous region, which makes it difficult to determine the genomic structure. To address this, we aimed to clarify the genomic structure of the *LILRA6* CNVs using the JoGo-LILR CN Caller, a tool engineered to delineate the genomic structure of *LILRB3* and *LILRA6* copy numbers from srWGS data. During this process, we unexpectedly discovered a novel large 33,692 bp deletion that results in the hybrid gene *LILRB5-LILRB3*.

## 2 Materials and methods

### 2.1 Ethics

The research ethics committee of Kanazawa University, Kyushu University, and National Center for Global Health and Medicine reviewed and approved this study. All participants provided written informed consent.

### 2.2 Samples

The original 2,504 unrelated samples from the 1kGP were used as a discovery cohort, and their srWGS data were downloaded from the International Genome Sample Resource website (https://www.internationalgenome.org/data-portal/data-collection/30x-grch38). Genomic DNA and RNA were obtained from the blood samples of volunteers belonging to Kanazawa University and residents of Shika town, where the cohort study was being conducted (24). Shika town is located on the Noto Peninsula in Ishikawa Prefecture, Japan. Total of 1,310 samples from an independent Shika cohort were used as a validation cohort in the Japanese population. Genomic DNA was extracted from the blood samples using the QIAamp DNA Blood Mini or Maxi Kit (Qiagen, Hilden, Germany), according to the manufacturer’s instructions, or extracted by SRL Inc. (Tokyo, Japan). Peripheral blood mononuclear cells (PBMCs) were isolated from blood samples by density gradient centrifugation using Ficoll-Paque PLUS (GE Healthcare, Pittsburgh, PA, USA). Subsequently, RNA was extracted from the PBMCs using the FastGene RNA Premium Kit (NIPPON Genetics, Tokyo, Japan) in accordance with the manufacturer’s instructions. First-strand cDNA synthesis was performed using a PrimeScript II 1^st^ strand cDNA Synthesis Kit (TaKaRa, Shiga, Japan) in accordance with the manufacturer’s instructions. Genomic DNA from the HapMap JPT samples was obtained from the Coriell Cell Repository. Among the three samples (NA12413, NA18959, and NA19087) identified with JoGo-LILR CN Caller, NA18959, and NA19087 were used for long-read sequencing technology and NA12413 was used for JoGo-LILR-trio analysis.

### 2.3 Long-read sequencing

DNA was extracted from the immortalized cell line of GM18959 and GM19087 obtained from the Coriell Cell Repository (Camden, NJ, USA; corresponding to NA18959 and NA19087, respectively). The DNA was subjected to long-read whole genome sequencing (lrWGS) on Sequel IIe (Pacific Biosciences (PacBio)) with the standard high-fidelity (HiFi) sequencing protocol. For the subreads generated in Sequel IIe, the CCS tool was used to generate unmapped HiFi BAM files. The unmapped BAM files were mapped to the GRCh38 reference assembly (without decoy and alt contigs) using Minimap2 (ver. 2.23) (25) and created aligned BAM files. For the aligned BAM file of each sample, the aligned HiFi reads from chr19: 54,180,000 to 54,280,000 were extracted. For NA18959 and NA19087, a total of 122 and 116 reads mapped to chr19: 54,180,000 to 54,280,000 (*LILRB5*-*LILRA6*-*LILRB3* region) were obtained, respectively. To examine the long deletions in the HiFi reads, the reads were re-aligned to the GRCh38 reference assembly using Minimap2 with the option “-ax splice:hq” (**Supplementary Figures 1**, **2**), which can capture long deletions. For local *de novo* assembly of the extracted reads, HiFiasm (ver. 0.16.1) was also applied (26). The assembled contigs were aligned by Minimap2 with the option “-r 500,40000 -ax map-hifi” to allow long deletion alignment. The re-aligned and *de novo* assembled results were inspected on Integrative Genomics Viewer (IGV) (ver. 2.16.2) to analyze the deletion haplotype and the base-pair resolution length (**Supplementary Figures 1**, **2**).

### 2.4 Validation of the 33,692 bp deletion

The breakpoint of the 33,692 bp deletion was verified using Sanger sequencing. Briefly, polymerase chain reaction (PCR) was performed on genomic DNA to amplify a 6,612 bp product spanning from *LILRB5* 3’UTR to *LILRB3* exon 13 using primers (*LILRB5* forward primer:5’-CCTGCACAGCTGAGTCCAGT-3’ and *LILRB3* reverse primer:5’-TTAGTCATCTTTGAGTCAGGTGAG-3’). The PacI (aataatttaattaa) and NotI (aataatgcggccgc) restriction sites were added to the 5’ sequences of the *LILRB5* forward and *LILRB3* reverse primers, respectively, to allow for gene cloning. Following treatment of the PCR products with ExoSAP-IT Express PCR Cleanup Reagents (Thermo Fisher Scientific, Waltham, MA, USA), direct sequencing was performed using specific primers (**Supplementary Table 1**). Sanger sequencing data were analyzed using Sequencher software (Gene Codes Corporation). Multiple sequence alignments were performed using the Clustal Omega software. The copy numbers of *LILRB3* and *LILRA6* were quantified using genomic DNA and a QIAcuity Digital PCR System (Qiagen, Hilden, Germany) in accordance with the manufacturer’s instructions. Digital PCR primer/probe sets were used as previously described (14). Individuals with a single copy of *LILRB3* were considered carriers of the *LILRB5-3* hybrid gene.

### 2.5 cDNA cloning

To identify the *LILRB5* isoforms and *LILRB5-3* hybrid gene, PCR was performed with cDNA templates using TaKaRa Ex Premier DNA Polymerase (TaKaRa, Japan) with the following primers: 5′-CCTGCACAGCTGAGTCCAGT-3′ for the *LILRB5* forward primer, and 5′-TTAGTCATCTTTGAGTCAGGTGAG-3′ for the *LILRB3* reverse primer. The PCR conditions consisted of initial denaturation at 94 °C for 1** **min, followed by 40 cycles of denaturation at 98 °C for 10 s, annealing at 62.5 °C for 15 s, and extension at 68 °C for 2 min using a VeritiPro Thermal Cycler (Thermo Fisher Scientific). For cDNA cloning, the PCR products were inserted into the pMXs-puro retroviral expression vector according to standard cloning methods. For PCR-direct sequencing, PCR products treated with ExoSAP-IT Express PCR Cleanup Reagents (Thermo Fisher Scientific) were subjected to Sanger sequencing. cDNA clones that were consistent with the PCR-direct sequencing data were regarded as the major isoforms.

### 2.6 *LILRB3* and *LILRA6* CN calling from srWGS using JoGo-LILR CN caller

The JoGo-LILR CN Caller was employed to extract *LILRB3*-*LILRA6* CNs from srWGS data. The JoGo-LILR CN Caller is available on the Japanese Open Genome and Omics Portal (https://jogo.csml.org/public/JoGo-LILR). This tool operates by processing BAM or CRAM files aligned to the GRCh38DH reference assembly. It then calculates normalized CNs for specific regions within the *LILRB3* and *LILRA6* genes (termed LILRB3core and LILRA6core) and their gene body regions (named LILRB3+LILRA6). The current implementation uses CNVnator (version 0.4.1) (27) to calculate the normalized CN values.

For the normalized CN values, the JoGo-LILR tool generated the *LILR* cluster plot with LILRB3+LILRA6 on the x-axis and LILRB3core**/**LILRA6core on the y-axis. For the plot, joint CN calling was applied after clustering to the dataset. The tool is particularly adept at stably calling CNs even in datasets with a small sample size, ranging from a single sample to several hundreds. For this purpose, the 1000 Genomes Project (1kGP) samples are typically employed as a background dataset, providing a comprehensive reference for comparison. This practice is instrumental in enhancing the accuracy and reliability of CN calling across diverse sample sizes.

For the detection of the *LILRB5-3* hybrid gene, JoGo-LILR was applied to CRAM files from 2,504 non-pedigree and two pedigree samples from the 1kGP (**Supplementary Table 2**) (28). To establish a Japanese CN profile for the cohorts from the southwest region of Japan (SW-JP) and northeast region of Japan (NE-JP), we analyzed 348 quality control (QC)-passed samples from the Tokyo healthy control dataset and 180 samples from volunteers at the National Hospital Organization Nagasaki Medical Center, Nagasaki, Japan, adhering to a cnv2 ratio QC metric of >0.85. In this process, the 1kGP samples were again utilized as a background reference for joint CN calling.

Finally, the visualization of the *LILRB3*-*LILRA6* genomic region by short-read mapping was conducted using IGV (ver. 2.6.2) (29).

### 2.7 Estimation of the allelic CN type using JoGo-LILR-trio

The JoGo-LILR-trio algorithm is a specialized tool used for accurately predicting *LILRB3*-*LILRA6* CN types in trio genotypes. It operates on a probabilistic model that assesses all potential combinations of these CN types in both parents. The primary objective of the JoGo-LILR-trio algorithm is to identify pairs of haploid *LILRB3*-*LILRA6* CN types that align with Mendelian inheritance patterns evident in their offspring. This algorithm is particularly adept at handling situations where multiple parental CN type combinations are plausible. In such cases, JoGo-LILR-trio employs a strategic approach to select the most likely combination that would result in the observed CN types in the children. In this study, the trio dataset comprising individuals NA12413, NA12412, and NA12485 was utilized.

### 2.7 Flow cytometry

To assess the cell surface expression of LILRB5, plasmids encoding *LILRB5* and the *LILRB5-3* hybrid gene were transiently co-transfected with green fluorescent protein (GFP) into 293T cells using PEI Max (Polyscience). GFP-positive cells were then identified by flow cytometry, as previously described (15). The cell surface expression of LILRB5 and LILRB5-3 was analyzed by flow cytometry using an anti-LILRB5 antibody (clone 395239, R&D Systems, Minneapolis, MN, USA) and MACSQuant 10 (Miltenyi Biotec, Germany).

## 3 Results

### 3.1 Estimation of a novel *LILRB3*-missing haplotype from 1kGP srWGS

**Figure 1:**
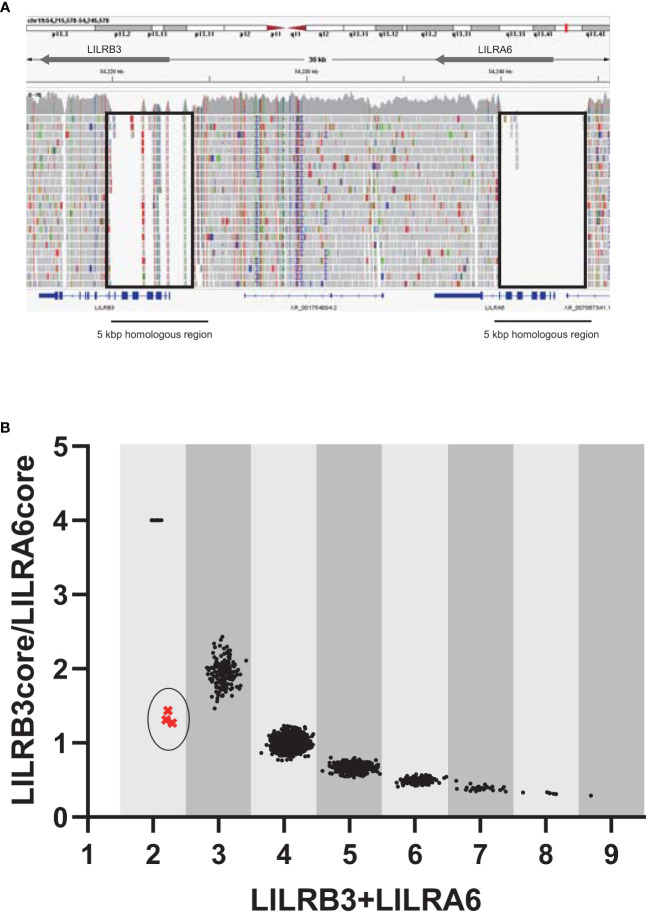
Estimation of the copy numbers within the *LILRB3-LILRA6* genomic region using pre-existing short-read sequencing data. **(A)** Visualization of short-read mapping to the *LILRB3-LILRA6* genomic region using the Integrative Genomics Viewer (IGV). One example of the individual NA18954 is shown. The block indicates the unmapped region with a low mapping quality (mapping quality threshold = 30). **(B)** Two-dimensional representation of *LILRB3* and *LILRA6* copy numbers (CN) in populations worldwide (n = 2,504). Each individual is represented by a single dot. The vertical and horizontal axes represent the *LILRB3*/*LILRA6* CN ratio and the sum of *LILRB3* and *LILRA6* copies, respectively. When *LILRA6* shows zero copy, the ratio of *LILRB3*/*LILRA6* becomes infinite. To visualize these dots on the graph, the *LILRB3*/*LILRA6* ratio corresponding to *LILRA6* zero copy was arbitrarily set to 4. The coordinate (x=2, y=4) indicates two copies of *LILRB3* in the absence of *LILRA6*. The circled cluster represents the individuals with the estimated haplotype that is missing *LILRB3*.

Since *LILRB3* and *LILRA6* exhibit a high degree of homology across a 5 kbp region encompassing the promoter and extracellular domains, usual analysis of srWGS cannot be properly mapped to a reference genome (**Figure 1A**). In contrast, the JoGo-LILR CN Caller called the *LILRB3*-*LILRA6* CNs with the *LILR* cluster plot and estimated the probable pair of allelic *LILRB3*-*LILRA6* CN type from srWGS (**Figure 1B**, **Supplementary Table 3**). As depicted in **Figure 1B**, nine distinct clusters were clearly illustrated in the two-dimensional representation of the sum of *LILRB3* and *LILRA6* CNs versus *LILRB3*/*LILRA6* CN ratios. The main clusters near the coordinates (3, 2), (4, 1), (5, 0.67), and (6, 0.5) indicate 1, 2, 3, and 4 copies of *LILRA6* with 2 copies of *LILRB3*, respectively. As noted in previous reports, the *LILRA6* copy number ranges from 0 to 6 in most individuals, with the exception of one individual who exhibited seven *LILRA6* copies (**Figure 1B**, **Supplementary Table 3**). The JoGo-LILR CN Caller also estimated the existence of a previously unreported *LILRB3*-missing haplotype in the three samples surrounding the coordinate (2, 1), NA12413, NA18959, and NA19087 with high probability (**Supplementary Table 4**).

### 3.2 Detection of a novel large 33,692 bp deletion in the *LILRB3*-*LILRA6* genomic region

**Figure 2:**
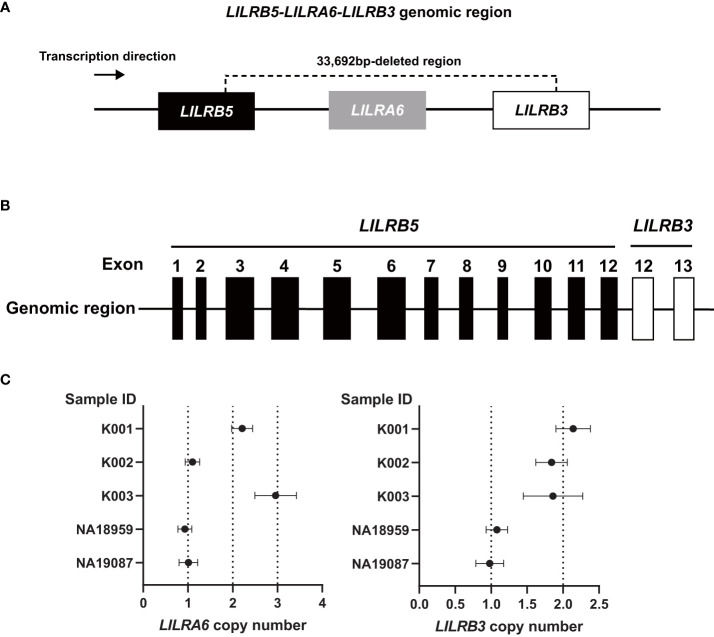
The 33,692 bp deletion removes *LILRB3* exons 1-11, the entire *LILRA6* gene, and *LILRB5* exon 13. **(A)** Schematic diagram of the 33,692 bp deletion. **(B)** The 33,692 bp deletion results in the *LILRB5-3* hybrid gene comprising *LILRB5* exons 1-12 and *LILRB3* exons 12-13. **(C)** *LILRA6* and *LILRB3* copy numbers of five individuals determined by digital PCR. The vertical and horizontal axes represent the sample ID and *LILRA6* copy number (left panel) or *LILRB3* copy number (right panel), respectively. The error bar indicates a 95% confidence interval. Individuals K001, K002, and K003 are examples of two, one, and three copies of *LILRA6*, respectively, while all three individuals possess two *LILRB3* copies. Carriers of the 33692 bp deletion (NA18959, and NA19087) possess a single copy of both *LILRA6* and *LILRB3*.

The analysis using srWGS is limited by only providing an overview of the structure of the *LILRB3* and *LILRA6* genomic region. For a more detailed examination at the base pair level, we employed real-time HiFi lrWGS on two Japanese-in-Tokyo (JPT) DNA samples, specifically NA18959 and NA19087. The lrWGS analysis revealed a novel large deletion (33,692 bp) carried by two individuals (**Supplementary Figures 1**, **2**). This 33,692 bp deletion spanned the *LILRB3*, *LILRA6*, and *LILRB5* genes (**Figure 2A**). This type of deletion is the largest ever reported and has not yet been detected in the *LILR* genomic region. Individuals carrying this deletion also had two copies of the *LILRA3* 6.7 kb deletion, indicating that the haplotype containing the 33,692 bp deletion has the fewest *LILR* genes. Thus, we carried out a detailed validation of the 33,692 bp deletion. After analyzing the region around the 33,692 bp deletion breakpoint using Sanger sequencing, we observed the presence of a 33,692 bp deletion eliminating exons 1–11 of *LILRB3*; *LILRA6*; and exon 13 of *LILRB5* (**Figure 2B**, **Supplementary Figure 3**). The 33,692 bp deletion left exons 1–12 of *LILRB5* and exons 12–13 of *LILRB3* intact, resulting in the hybrid gene structure of *LILRB5* and *LILRB3* (hereafter referred to as “*LILRB5-3*”). Examination of exon 8 of both *LILRB3* and *LILRA6* by digital PCR revealed that two individuals (NA18959 and NA19087) carrying the 33,692 bp deletion lacked a single copy of both genes, which was consistent with the sequence data (**Figure 2C**). These data suggest that the 33,692 bp deletion results in the hybrid gene *LILRB5-3* and the deletion of *LILRB3* and *LILRA6*.

### 3.3 Validation of the hybrid gene *LILRB5-3* in the independent Japanese cohort

To validate the existence of the hybrid gene *LILRB5-3* in the other Japanese samples, we subjected 1,310 samples from an independent Shika cohort study in Japan to digital PCR analysis (**Supplementary Table 5**) using the DNA extracted from whole blood. The analysis detected a single copy of both *LILRB3* and *LILRA6* in four samples. These results confirmed that the existence of the large deletion did not originate from the structural variation acquired in the lymphoblastoid cell lines in 1KGP.

### 3.4 Frequencies of *LILRB5-3* hybrid gene in the Japanese population

To ascertain the frequencies of the *LILRB5-3* hybrid gene within the Japanese population, we conducted an analysis using the JoGo-LILR CN Caller. Our dataset included 180 srWGS samples from SW-JP and 348 samples from NE-JP (**Supplementary Figures 4**, **5**, **Supplementary Tables 6, 7**). Among these, two samples from NE-JP were identified as carrying the hybrid gene, while none from SW-JP exhibited this genetic feature. Notably, the Japanese samples in the 1kGP project was also catalogued in NE-JP (Tokyo).

### 3.5 Frequencies of *LILRB5-3* hybrid gene in the Korean population

The presence of the *LILRB5-3* hybrid gene has been definitively confirmed in the Japanese population. This genetic feature was not detected in the other East Asian samples from the 1kGP, which included 93 CDX (Dai Chinese), 103 CHB (Han Chinese), 105 CHS (Southern Han Chinese), and 99 KHV (Kinh Vietnamese) individuals (**Supplementary Table 3**). However, the Korean population was not represented in the 1kGP dataset. Therefore, we examined 70 QC srWGS data from the Korean Personal Genome Project (KPGP) using JoGo-LILR CN Caller (**Supplementary Figure 6**, **Supplementary Table 8**). Our analysis revealed the absence of the *LILRB5-3* hybrid gene in these Korean samples. Since the *LILRB5-3* hybrid gene appears to be rare in the Japanese population, additional sequencing is needed to definitively determine its presence or absence in the other regions of East Asia.

### 3.6 *LILRB5-3* hybrid gene in other populations

Among a total of 2,504 unrelated samples analyzed, only one individual, NA12413 from the CEU cohort of European descent, was identified with a single CN for both *LILRA6* and *LILRB3*, aside from two samples in the JPT cohort. This individual, NA12413, along with NA12412 and NA12485, were annotated as a father, mother, and child, respectively. All these srWGS data were available as pedigree dataset in 1kGP project (28). Utilizing the JoGo-LILR-trio tool, which is designed to predict the most likely pair of allelic *LILRB3*-*LILRA6* CN types in offspring, we analyzed this trio’s srWGS data. The analysis revealed that the child, NA12485, possesses one copy of both *LILRB3* and *LILRA6* genes and is estimated to have a genotype with zero copies of both *LILRB3*-*LILRA6* on one allele and a single copy of both *LILRB3*-*LILRA6* on the other allele (JoGo-LILR CN Caller value (x=2.3, y=1.4)). This observation suggests that the *LILRB5-3* hybrid gene was transmitted from the father (NA12413) to the child (NA12485), and provides robust support for the existence of the hybrid gene in the CEU population.

### 3.7 Validation of the expression of the hybrid gene *LILRB5-3*

**Figure 3:**
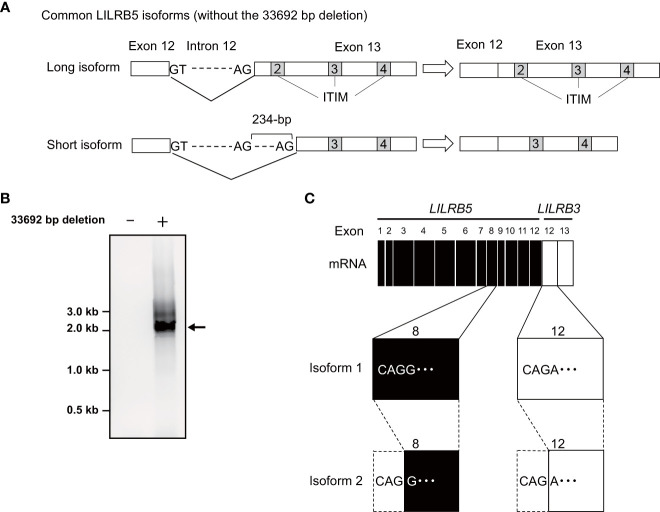
Identification of the *LILRB5* and *LILRB5-3* hybrid isoforms. **(A)** A schematic diagram of long and short isoforms of *LILRB5* identified from non-carriers of the 33692 bp deletion. The short isoform lacks 234-bp of exon 13 containing a second immunoreceptor tyrosine-based inhibitory motif (ITIM), compared with the long isoform. **(B)** An RT-PCR analysis, utilizing a forward primer positioned at *LILRB5* exon 1 and a reverse primer positioned at *LILRB3* exon 13, amplified the expected size of the *LILRB5-3* hybrid gene in the carrier (indicated as +), but not in the non-carrier (indicated as -). The arrow indicates the expected PCR product of the *LILRB5-3* hybrid gene. **(C)** cDNA cloning of the PCR products identified two alternatively spliced isoforms of the *LILRB5-3* hybrid gene.

**Figure 4:**
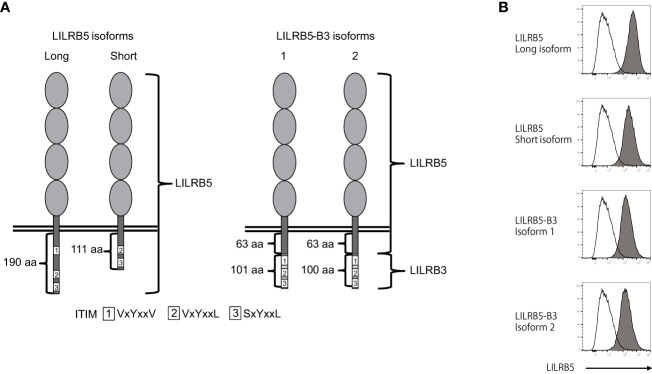
The LILRB5-3 hybrid can be expressed at the protein level. **(A)** A schematic diagram of the protein products of long and short *LILRB5* isoforms in addition to LILRB5-3 isoforms 1 and 2. The four elliptical structures in the extracellular region indicate Ig-like domains. The aa indicates amino acid. **(B)** Plasmids encoding the full-length of long and short LILRB5 isoforms, as well as LILRB5-3 isoforms 1 and 2, were transiently co-transfected with green fluorescent protein (GFP) into 293T cells. The transfectants were subsequently stained with an anti-LILRB5 antibody (clone 395239), and the GFP-positive cells were analyzed. Open and closed histograms represent mock and LILRB5 transfectants, respectively. A shift of cell populations from the left (open histogram) to the right (closed histogram) indicates cell surface expression of LILRB5 and LILRB5-3.

To verify the expression of the *LILRB5-3* hybrid gene, PCR-direct sequencing and cDNA cloning were conducted on Japanese individuals with and without the 33,692 bp deletion. Two primary *LILRB5* isoforms, long and short, were identified in individuals without deletions through cDNA cloning of *LILRB5* transcripts. The long isoform contained three ITIMs, whereas the short isoform contained two ITIMs (**Figure 3A**). This difference is due to alternative splicing of the splice acceptor site of intron 12. In contrast, a chimeric gene composed of *LILRB5* and *LILRB3* was detected in a Japanese individual with a deletion using *LILRB5* forward and *LILRB3* reverse primers (**Figure 3B**). The amplicon was approximately 2 kb in size, which was consistent with the expected size of the exonic region of *LILRB5-3* (**Figure 3B**). cDNA cloning and sequencing identified two isoforms of a chimeric gene composed of *LILRB5* and *LILRB3*, providing evidence that *LILRB5-3* was transcribed at the mRNA level (**Figure 3C**). These two isoforms differ by three bp at the acceptor sites of exons 8 and 12 of *LILRB5* and *LILRB3*, respectively. This resulted in a disparity between the two amino acids that were not located within any functional domains or motifs. The hybrid junction between LILRB5 and LILRB3 was located within the intracellular domain and fused the extracellular domain of LILRB5 with the intracellular domains containing partial LILRB3. The original number of ITIMs in LILRB3 was four, whereas LILRB5-3 contained three of the four LILRB3 ITIMs (**Figure 4A**). This suggests that LILRB5-3 acquired a novel signaling pathway.

There exists an example wherein the killer cell Ig-like receptor, KIR3DL1*004, lacks cell surface expression but exhibits intracellular retention (30). Therefore, we investigated whether LILRB5-3 can be displayed on the cell surface. To this end, we transfected the identified *LILRB5* and *LILRB5-3* genes into 293T cells and stained the transfectants with an anti-LILRB5 antibody. Flow cytometry confirmed the detection of the LILRB5-3 proteins on the cell surface, demonstrating that LILRB5-3 can be expressed at the cell-surface protein level (**Figure 4B**). These data suggest that LILRB5-3 may have a novel function in carriers with the 33,692 bp deletion.

## 4 Discussion

**Figure 5:**
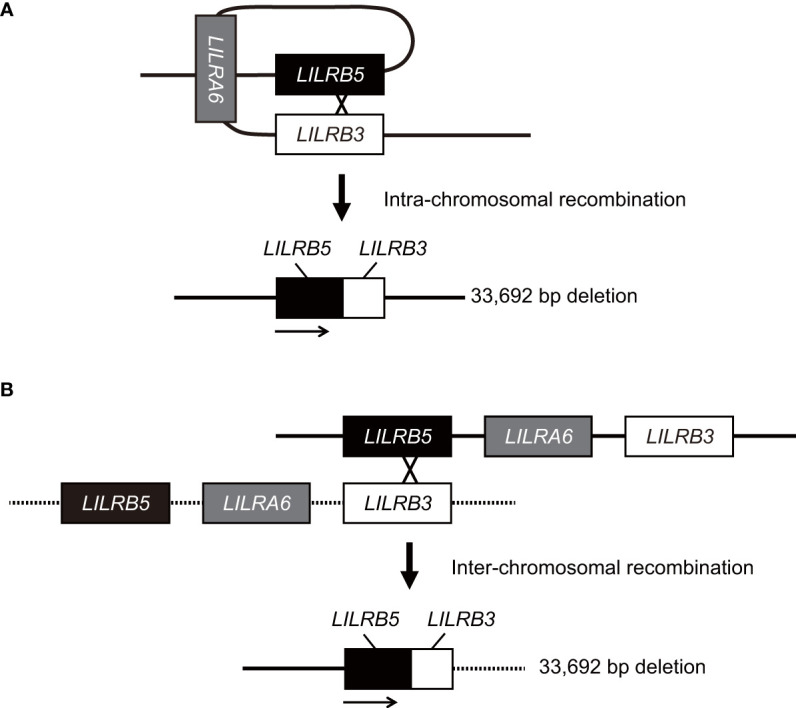
Model for the generation of the *LILRB5-3* hybrid gene. The *LILRB5-3* hybrid gene may have been generated by the non-allelic homologous recombination of misaligned *LILRB3* and *LILRB5* during meiosis. Two putative mechanisms that underlie the *LILRB5-3* hybrid gene are intra- **(A)** and inter-chromosomal homologous recombination **(B)**. Solid and dashed lines indicate one, and another chromosomes, respectively. Intra-chromosomal recombination preserves the identical haplotype.

In this study, we identified a novel large 33,692 bp deletion in the *LILRA6* genomic region using long-read sequencing technology. The 33,692 bp deletion, which lacks both *LILRB3* and *LILRA6*, is the largest reported deletion within the *LILR* genomic region. The 33,692 bp deletion was unique in generating the hybrid gene of *LILRB5* and *LILRB3*. It is estimated that the breakpoints of the 33,692 bp deletion are located within intron 12 of the *LILRB5* gene and intron 11 of the LILRB3 gene (**Supplementary Figure 3**). These two introns share a high degree of homology, with 269 base pairs of complete identity. These observations suggest that non-allelic homologous recombination occurred at this site. Similar hybrid genes are also found within the killer cell Ig-like receptor (*KIR*) gene cluster, which lies in close proximity to the *LILR* region on chromosome 19 (31). In particular, the hybrid gene between *KIR2DL1* and *KIR3DL2* has arisen by a deletion spanning the intermediate and telomeric regions (32). The region of recombination encompasses a 129 bp sequence, which is identically found in both *KIR2DL1* and *KIR3DL2* alleles, a similar pattern observed in *LILRB5-3*. However, the limitation of this study is that the precise mechanism underlying the 33,692 bp deletion remains uncertain. Specifically, whether the 33,692 bp deletion results from intra- or inter-chromosomal recombination remains elusive due to the lack of the haplotype data around the *LILRB5*-*LILRA6*-*LILRB3* genomic region in human populations (**Figure 5**). Haplotyping of these regions would provide valuable insights into the potential mechanisms driving this substantial genetic alteration.

Recently, a thorough comparative analysis between the primate *LILR* genomic loci was conducted, revealing that the *LILR* region remained largely conserved throughout primate evolution (33). However, the *LILRA6* ortholog was lacking in orangutans and common marmosets. Due to limited sample sizes, it is currently unclear whether the absence of *LILRA6* in orangutans and common marmosets represents CNV. Future analyses of non-human primates will uncover the evolutionary dynamics of the *LILR* CNVs in primates.

The presence of the *LILRB5-3* hybrid transcript was confirmed in an individual carrying the hybrid gene but was not detected in a non-carrier. The *LILRB5-3* hybrid gene produces a protein that contains the extracellular and transmembrane domains of LILRB5, a fragment of the LILRB5 intracellular region devoid of ITIMs, and a portion of the LILRB3 intracellular region that contains the second, third, and fourth ITIMs of LILRB3. This suggests that the hybrid receptor recognizes ligands through the LILRB5 extracellular domain, whereas receptor signaling is mediated by previously unobserved motifs. Although HLA class I-free heavy chains have been reported to be ligands for LILRB5, the physiological significance of this interaction is still not well understood (34). In contrast, the novel signaling motif of the hybrid gene, which contains three instead of four LILRB3 ITIMs, may represent a new function. The intracellular domain of LILRB3 can be divided functionally into two distinct regions: one responsible for binding TRAF2 and the other for binding SHP-1 (35). The intracellular domain of LILRB5-3 lacks the TRAF2 binding site of LILRB3 due to replacement by the LILRB5 intracellular domain. In contrast, the LILRB5-3 intracellular domain still retains the SHP-1 binding site of LILRB3. Consequently, LILRB5-3 shows a new hybrid motif that combines the LILRB5 and LILRB3 intracellular regions. Therefore, the function of the hybrid gene needs to be confirmed experimentally.

We have applied JoGo-LILR CN Caller to call *LILRA6* CNs from pre-existing srWGS data. The tool was capable of accurately detecting not only the candidate 33,692 bp deletion that contains the *LILRB5-3* hybrid gene, but also multiple copies of *LILRA6* in populations worldwide. Therefore, the tool is a useful tool for disease-association studies. Individuals with the 33,692 bp deletion were more frequent in the Japanese population than in other populations worldwide. Genome-wide association studies have indicated that the *LILRB5* SNP is significantly associated with creatine kinase levels (17, 18), and therefore, the 33,692 bp deletion may also be linked to a particular phenotype in the Japanese population. In addition, the haplotype of the *LILRB5-3* hybrid gene also lacks *LILRA3*. Since *LILRA3* deletion is associated with Takayasu arteritis in the Japanese population (21), it is conceivable that the combination of the *LILRB5-3* hybrid gene and the *LILRA3* deletion may be useful for further classification of the pathogenesis of Takayasu arteritis.

Given that the region with high homology between *LILRB3* and *LILRA6* is approximately 5 kb in length, it is difficult to distinguish between *LILRB3* and *LILRA6* using SNP-based genotyping and short-read sequencing technology. We previously showed that 1 KG data contains a significant number of genotyping errors due to the utilization of short-read sequencing technology (15). Therefore, regions with high homology will become increasingly accessible using long-read sequencing technology. The deleted fragment contains both *LILRB3* and *LILRA6*, which are paired inhibitory and activating receptors, respectively. However, it remains unclear how they function cooperatively. Future functional studies and large-scale disease association studies will provide insight into the immunological significance of this large deletion within the human population.

## Data availability statement

The data presented in the study are deposited in the DDBJ/EMBL/GenBank repository, accession number LC785381 (LILRB5 long isoform); LC785382 (LILRB5 short isoform); LC785383 (LILRB5-3 isoform 1); LC785384 (LILRB5-3 isoform 2); and LC785385 (LILRB5-3 genomic sequence).

## Ethics statement

The studies involving humans were approved by the research ethics committee of Kanazawa University, Kyushu University, and National Center for Global Health and Medicine. The studies were conducted in accordance with the local legislation and institutional requirements. The participants provided their written informed consent to participate in this study.

## Author contributions

KHi: Conceptualization, Data curation, Formal analysis, Funding acquisition, Investigation, Methodology, Project administration, Resources, Software, Supervision, Validation, Visualization, Writing – original draft, Writing – review & editing. S-SK: Conceptualization, Investigation, Methodology, Project administration, Resources, Software, Writing – original draft, Writing – review & editing. YK: Investigation, Methodology, Project administration, Resources, Software, Writing – original draft, Writing – review & editing. MS: Data curation, Methodology, Resources, Writing – original draft, Writing – review & editing. YO: Data curation, Methodology, Resources, Writing – original draft, Writing – review & editing. GH: Investigation, Writing – original draft, Writing – review & editing. YH: Investigation, Writing – original draft, Writing – review & editing. HT: Investigation, Writing – original draft, Writing – review & editing. JO: Resources, Writing – original draft, Writing – review & editing. KHo: Resources, Writing – original draft, Writing – review & editing. AT: Resources, Writing – original draft, Writing – review & editing. HN: Resources, Writing – original draft, Writing – review & editing. MiN: Resources, Writing – original draft, Writing – review & editing. KT: Writing – original draft, Writing – review & editing, Project administration, Resources, Supervision. RH: Project administration, Resources, Supervision, Writing – original draft, Writing – review & editing. MaN: Conceptualization, Data curation, Formal analysis, Funding acquisition, Investigation, Methodology, Project administration, Resources, Software, Supervision, Validation, Visualization, Writing – original draft, Writing – review & editing.
