# Supplementary material for: Identification of the hybrid gene LILRB5-3 by long-read sequencing and implication of its novel signaling function
Source: Front Immunol. 2024 May 14;15:1398935. doi: 10.3389/fimmu.2024.1398935 (PMC11130398; doi:10.3389/fimmu.2024.1398935)
Supplement: Supplementary file 1 [file DataSheet_1.pdf]

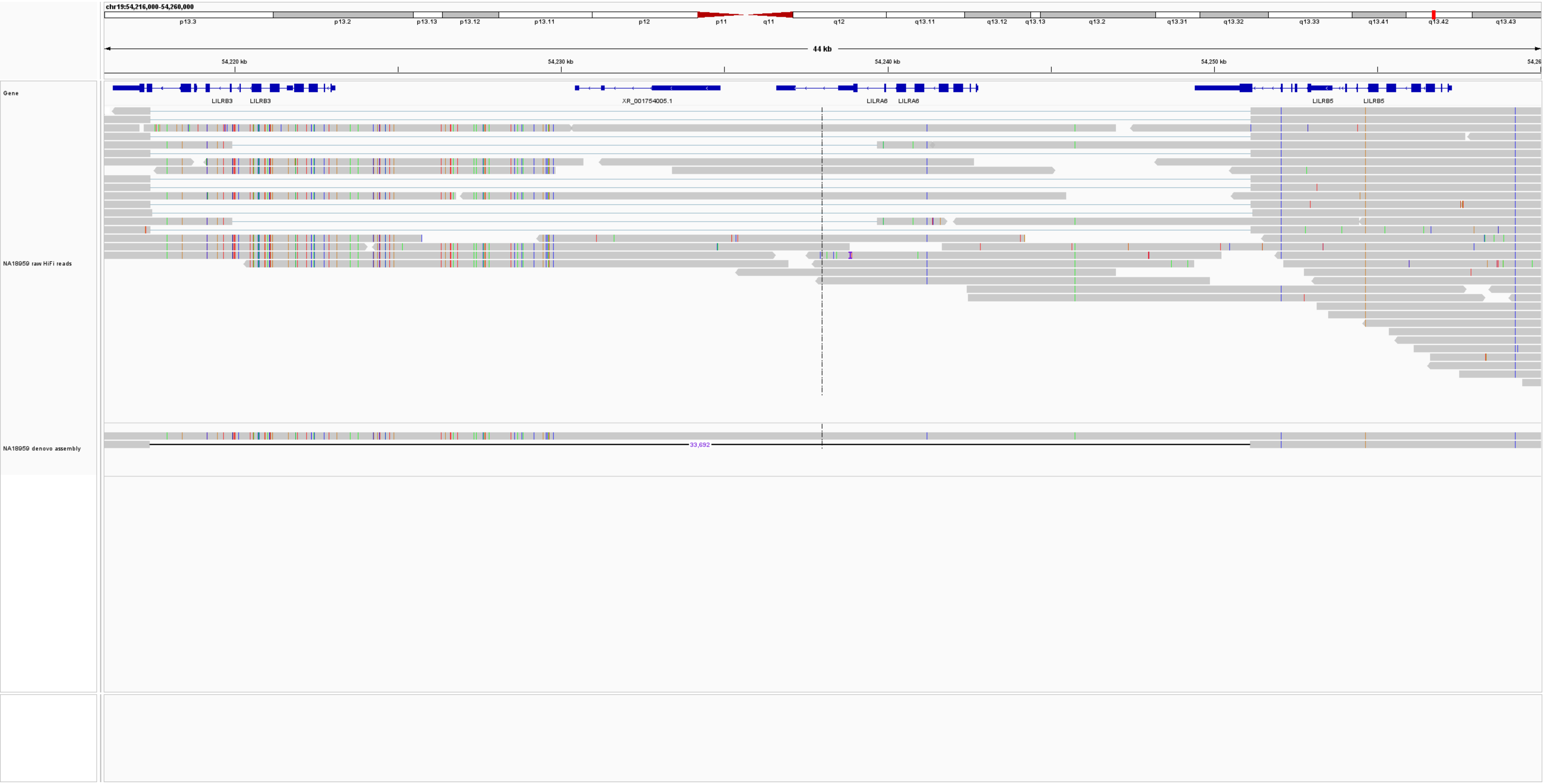

Supplementary Figure 1 The iGV snapshot of NA18959 sample with the 33,692 bp deletion. The top is the HiFi sequencing reads and the bottom is the de novo assembled result.

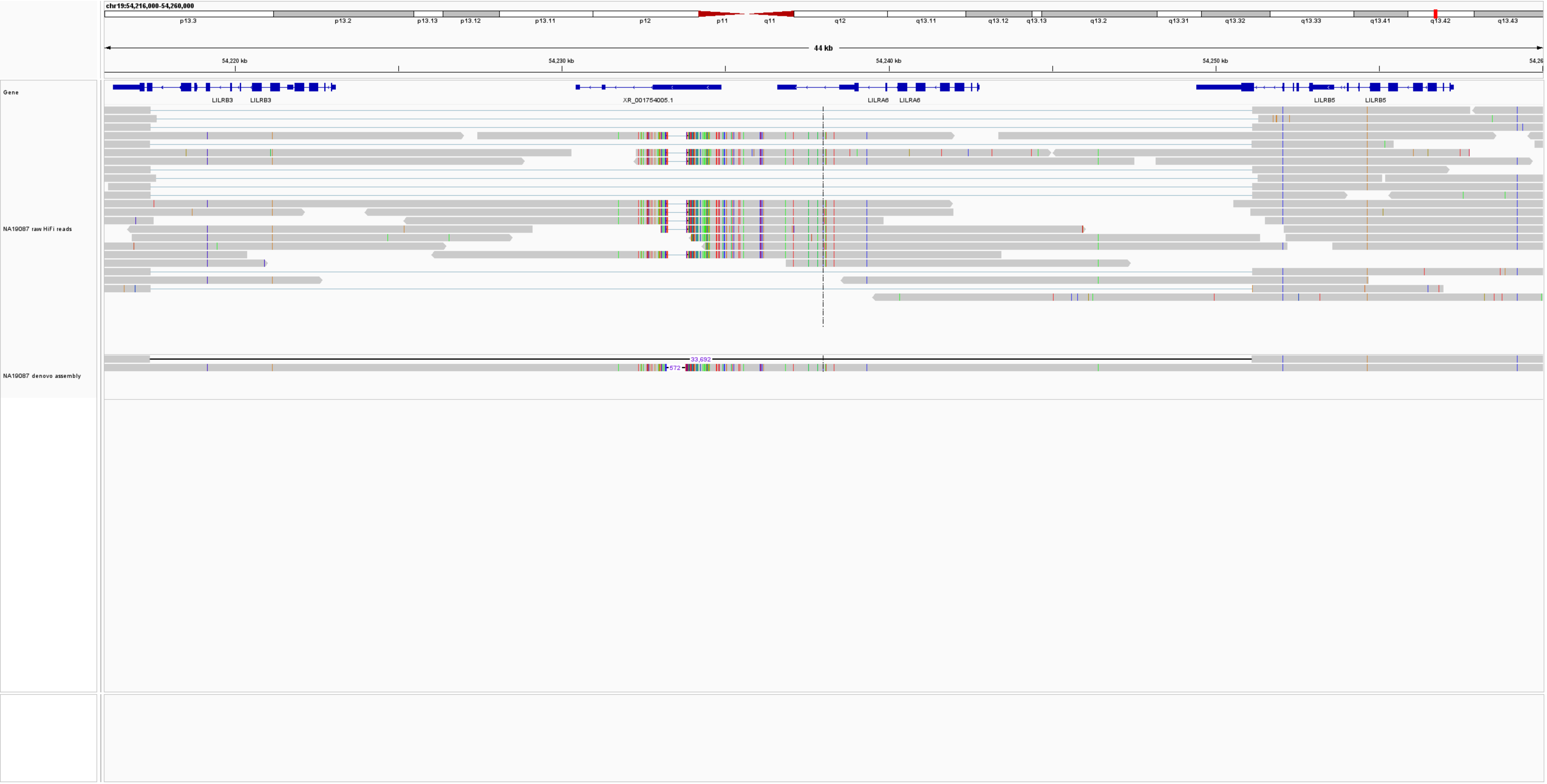

Supplementary Figure 2 The iGV snapshot of NA19087 sample with the 33,692 bp deletion. The top is the HiFi sequencing reads and the bottom is the de novo assembled result.

	LILRB3 exon 11/LILRB5 exon 12
LILRB3	ATGCTGCTGTGAAGGACACACAGTCTGAGGACAGGGTGGAGCTGGACAGTCAGGTGAGAT
LILRB5-3	ATGCTGCCGTGAAGGACACACAGCCCAAGGACGGGGTGGAGATGGATGCTCGGGTGAGGC
LILRB5	ATGCTGCCGTGAAGGACACACAGCCCAAGGACGGGGTGGAGATGGATGCTCGGGTGAGGC

	LILRB3 intron 11/LILRB5 intron 12
LILRB3	CCC-GCCCCGTCCCAGGCACCAAAGGCCTCCTGGTGCCAGATCTAATCCTGCAGGACTTC
LILRB5-3	CCCGCCCCTGTCCCGGGCACCAAAGGCCTCCTGGTGCCAGATCTAATCCAGCAGGACTTC
LILRB5	CCCGCCCCTGTCCCGGGCACCAAAGGCCTCCTGGTGCCAGATCTAATCCAGCAGGACTTC

	LILRB3 intron 11/LILRB5 intron 12
LILRB3	TCTGTCCTCCTTCCCCCGGCTCTCAGCATCGTCACGGTGGACCCCTCCTTGTCCAGCATG
LILRB5-3	TCTGTCCTCCTTCCCCCGGCTCTCAGCATCGTCACGGTGGACCCCTCCTTGTCCAGCACG
LILRB5	TCTGTCCTCCTTCCCCCGGCTCTCAGCATCGTCACGGTGGACCCCTCCTTGTCCAGCACG

	LILRB3 intron 11/LILRB5 intron 12
LILRB3	CTGCCTCCCGCCTGCTGTGACCTCACTCTCTTCTGCTGTCCTGGGACCTCGTGGGCCTCC
LILRB5-3	CTGCCTCCCGCCTGCTGCGACCTCACTCTCTTCTGCTGTCCTGGGACCTCGTGGGCCTCC
LILRB5	CTGCCTCCCGCCTGCTGCGACCTCACTCTCTTCTGCTGTCCTGGGACCTCGTGGGCCTCC

	LILRB3 intron 11/LILRB5 intron 12
LILRB3	TCCCGGGTCCCCTTCCTGCTCCTCATCCTCTGTTTGGCCGTCTGGTTGTTAGAGCTCTCC
LILRB5-3	TCCCGGGTCCCCTTCCTGCTCCTCATCCTCTGTTTGGCCGTCTGGTTGTTAGAGCTCTCC
LILRB5	TCCCGGGTCCCCTTCCTGCTCCTCATCCTCTGTTTGGCCGTCTGGTTGTTAGAGCTCTCC

	LILRB3 intron 11/LILRB5 intron 12
LILRB3	CCAGGCCTCAGGAGGATGACGAATAAATGAACCACCTCCGTCCCCTGGGCTCCTCTTCAT
LILRB5-3	CCAGGCCTCAGGAGGATGAGGAATAAATGAACCGACCCGGTCCCCCAGGCTCCCCTTCAT
LILRB5	CCAGGCCTCAGGAGGATGAGGAATAAATGAACCGACCCGGTCCCCCAGGCTCCCCTTCAT

	LILRB3 intron 11/LILRB5 intron 12
LILRB3	TCATTCATCCAGCGAGTGTTCCCAGGGAGCTCACTGTGGATGGGGCTCCCCATGGGAGCT
LILRB5-3	TCATTCAACCAGCGAGTGTTCCCAGGGAGCTCACTGTGGATGGGGCTCCCCATGGGAGCT
LILRB5	TCATTCAACCAGCGAGTGTTCCCAGGGAGCTCACTGTGGATGGGGCTCCCCATGGGAGCT

	LILRB3 intron 11/LILRB5 intron 12
LILRB3	GCAGACACAGCAGGGAGCAAAGCCGCCCCCGCCTCCTGAGCTCACCTCACGGTGGGAGAC
LILRB5-3	GCAGACACAGCAGGGAGCAAAGCCGCCCCCGCCTCCTGAGCTCACCTCATGGTGGGAGAC
LILRB5	GCAGACACAGCAGGGAGCAAAGCCGCCCCCGCCTCCTGAGCTCACCTCATGGTGGGAGAC

	LILRB3 intron 11/LILRB5 intron 12
LILRB3	AAAATGCAAATAAATGCATCGTGTCCAGGAGTGCAACGTGCTGTAAGGAACATACACCAG
LILRB5-3	AAAATGCAAATAAATGCATCGTGTCCAGGAGTGCAACGTGCTGTAAGGAACATAAACCAG
LILRB5	AAAATGCAAATAAATGCATCGTGTCCAGGAGTGCAACGTGCTGTAAGGAACATAAACCAG

	LILRB3 intron 11/LILRB5 intron 12
LILRB3	GGAAAGGGCAGAGAGTGTGGGGCAGTGGGGCCAGTCTGAATGGAAGGGGAGGGCTGTCTG
LILRB5-3	GGAAAGGGCAGAGAGTGTGGGGCAGTGGGGCCAGTCTGAATGGAAGGGGAGGGCTGTCTG
LILRB5	GGAAAGGGCAGAGAGTGTGGGGCAGTGGGGCCAGTCTGAATGGAAGGGGAGGGCTGTCTG

	LILRB3 intron 11/LILRB5 intron 12
LILRB3	CTCAGCTGTCATCTGAGAAGCCTGGACAGAGTGGGGCACATGATCCTCTGATGGACGAGC
LILRB5-3	CTCAGCTGTCATCTGAGAAGCCTGGACAGAGTGGGGCACACGATCCTCTGATGGACGAGC
LILRB5	CTCAGCTGTCATCTGAGAAGCCTGGACAGAGTGGGGCACACGATCCTCTGATGGACGAGC

	LILRB3 intron 11/LILRB5 intron 12
LILRB3	CCCTGCAGGCAGAGGAAACAGCCGTGCAAAGGCCCCCAGGCAGCAGCGAGCTCTTGCAGG
LILRB5-3	CCCTGCAGGCAGAGGAAACAGCCGTGCAAAGGCCCCGAGGCAGCAGCGAGCTCTTGCGGG
LILRB5	CCCTGCAGGCAGAGGAAACAGCCGTGCAAAGGCCCCGAGGCAGCAGCGAGCTCTTGCGGG

	LILRB3 intron 11/LILRB5 intron 12
LILRB3	AAGGCCTGTGAGGCTGCAGCCAAATGGGCAAGGTCAGAGTGAGGAGCAGAGGCCAGAACC
LILRB5-3	AAGGCCCGTGAGGCTGCAGCCAAATGGGCAAGGTCAGAGTGAGGAGCAGAGGCCAGAACC
LILRB5	AAGGCCCGTGAGGCTGCAGCCAAATGGGCAAGGTCAGAGTGAGGAGCAGAGGCCAGAACC

	LILRB3 intron 11/LILRB5 intron 12
LILRB3	ACAGGGAGGGAGCGGCCAGACCCTCCACGGCCTTAGGGCGTCCCTGAGATTCCATCAGGA
LILRB5-3	ACAGGGAGGGAGCGGCCAGACCCTCCACGGCCTTAGGGCGTCCCTGAGATTCCATCAGGA
LILRB5	ACAGGGAGGGAGCGGCCAGACCCTCCACGGCCTTAGGGCGTCCCTGAGATTCCATCAGGA

	LILRB3 intron 11/LILRB5 intron 12
LILRB3	AAGGGATGTAATCGGATCACCCCGGGAACAGTGAGGAAAATTGACTCCAGGAGGTCAGGG
LILRB5-3	AAGGGATGTAATCGGATCACCCCGGGAACAGTGAGGAAAATTGACTCCAGGAGGTCAGGG
LILRB5	AAGGGATGTAATCGGATCACCCCGGGAACAGTGAGGAAAATTGACTCCAGGAGGTCAGGG

	LILRB3 intron 11/LILRB5 intron 12	LILRB3 exon 12/LILRB5 exon 13
LILRB3	GGACTCAAGGACACCCCCCACCACTGTCTCTCTCCAG	CAGAGCCCACACGATGAAGACCC
LILRB5-3	GGACTCAAGGACACCCCCCACCACTGTCTCTCTCCAG	CAGAGCCCACACGATGAAGACCC
LILRB5	GGACTCAAGGACACCCCCCACCACTGTCTCTCTCCAG	CAGAGCCCACACGATGAAGACCC

	LILRB3 exon 12/LILRB5 exon 13
LILRB3	CCAGGCAGTGACGTATGCCCCGGTGAAACACTCCAGTCCTAGGAGAGAAATGGCCTCTCC
LILRB5-3	CCAGGCAGTGACGTATGCCCCGGTGAAACACTCCAGTCCTAGGAGAGAAATGGCCTCTCC
LILRB5	CCAGGCAGTGACGTATGCCCCGGTGAAACACTCCAGACCTAGGAGAGAAATGGCCTCTCC

	LILRB3 exon 12/LILRB5 exon 13
LILRB3	TCCCTCCTCACTGTCTGGGGAATTCCTGGACACAAAGGACAGACAGGTGGAAGAGGACAG
LILRB5-3	TCCCTCCTCACTGTCTGGGGAATTCCTGGACACAAAGGACAGACAGGTGGAAGAGGACAG
LILRB5	TCCTTCCCCACTGTCTGGGGAATTCCTGGACACAAAGGACAGACAGGCAGAAGAGGACAG

	LILRB3 exon 12/LILRB5 exon 13
LILRB3	GCAGATGGACACTGAG
LILRB5-3	GCAGATGGACACTGAG
LILRB5	ACAGATGGACACTGAG

Supplementary Figure 3 Sequence alignment of *LILRB3* , *LILRB5-3* , and *LILRB5*

Sequence alignment around the hybrid junction is indicated. The base pairs mismatched with *LILRB5-3* are gray-colored.

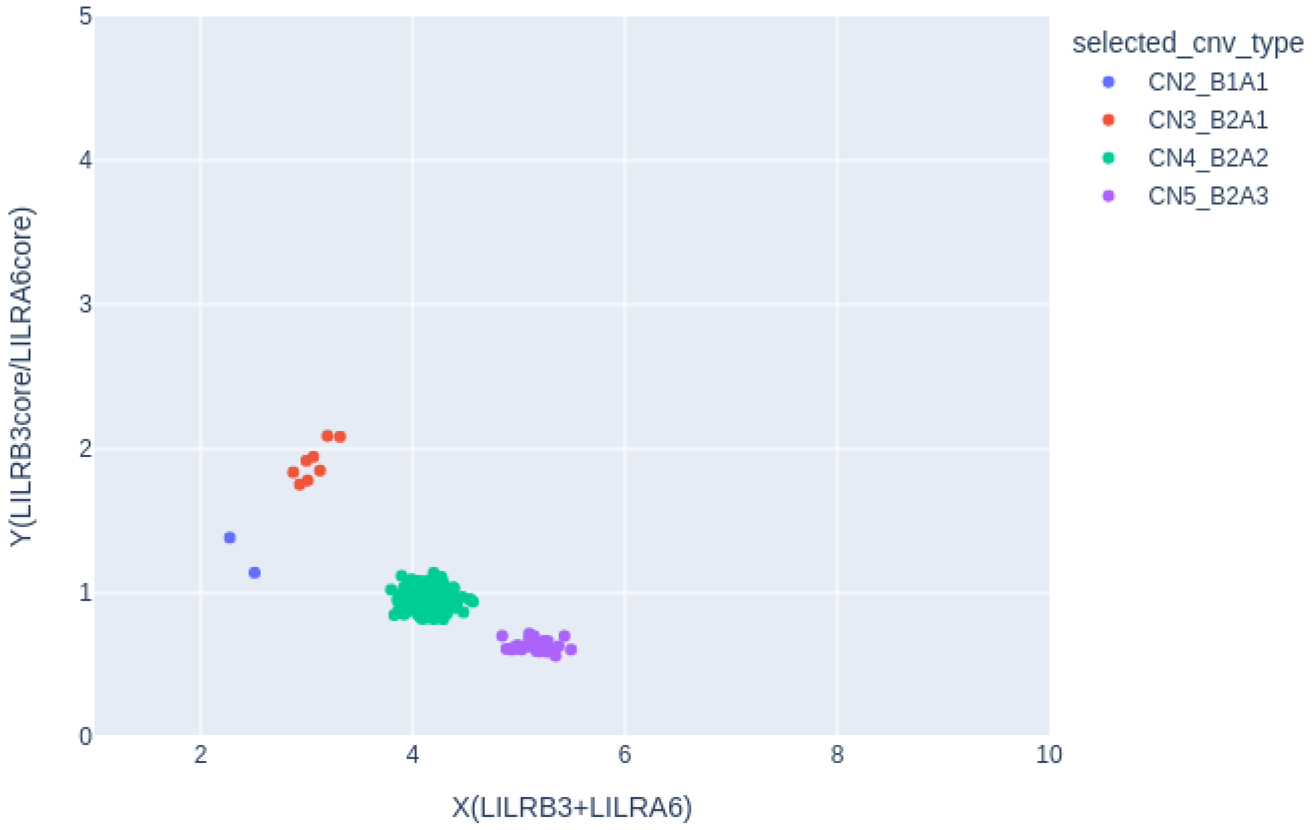

Supplementary Figure 4 The clustering plot of JoGo-LILR to 348 srWGS samples in the northeast region in Japan.

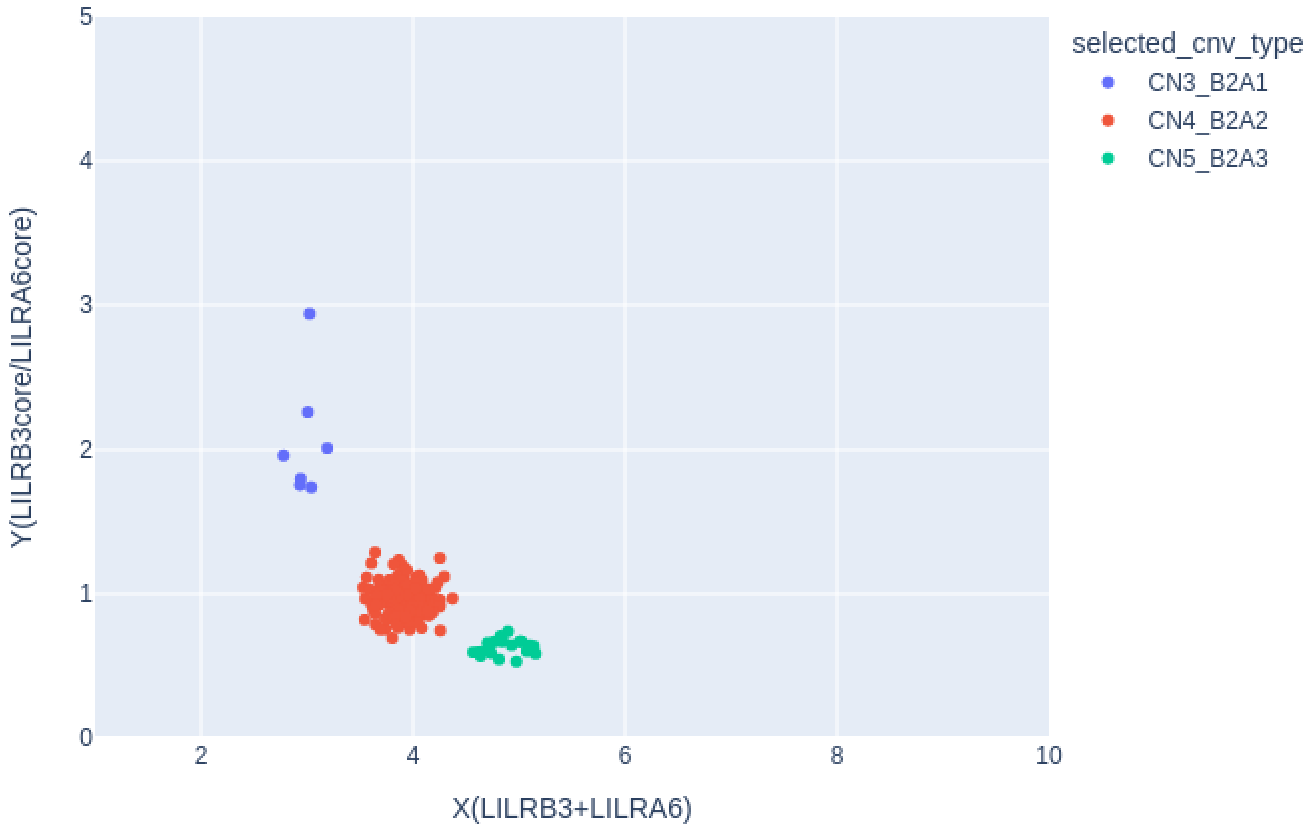

Supplementary Figure 5 The clustering plot of JoGo-LILR to 180 srWGS samples in the southwest region in Japan.

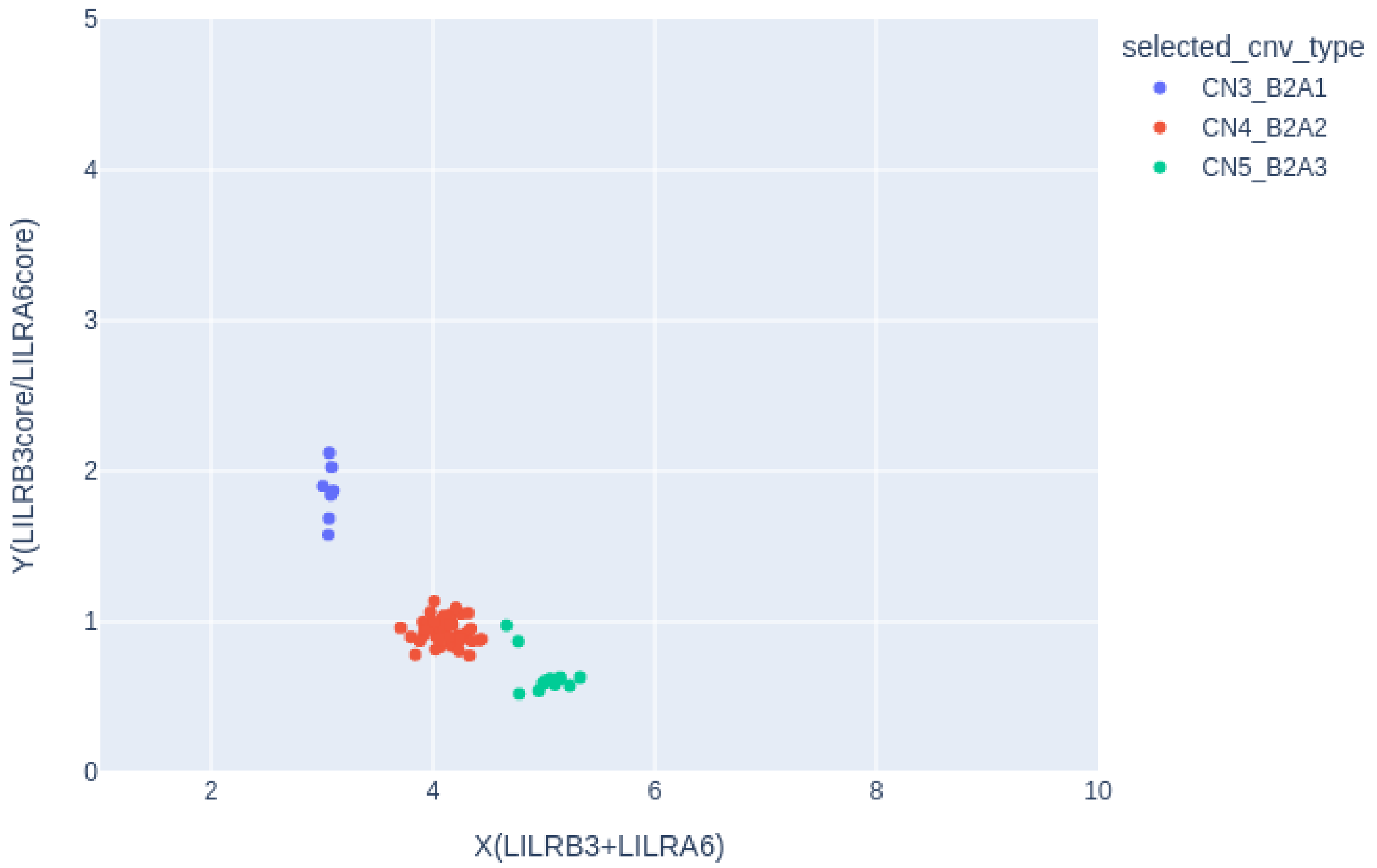

Supplementary Figure 6 The clustering plot of JoGo-LILR to to 70 srWGS samples in Korea.
